# The peptidylarginine deiminase gene is a conserved feature of *Porphyromonas gingivalis*

**DOI:** 10.1038/srep13936

**Published:** 2015-09-25

**Authors:** Giorgio Gabarrini, Menke de Smit, Johanna Westra, Elisabeth Brouwer, Arjan Vissink, Kai Zhou, John W. A. Rossen, Tim Stobernack, Jan Maarten van Dijl, Arie Jan van Winkelhoff

**Affiliations:** 1 Center for Dentistry and Oral Hygiene, University of Groningen and University Medical Center Groningen, the Netherlands; 2 Department of Rheumatology and Clinical Immunology, University of Groningen and University Medical Center Groningen, the Netherlands; 3 Department of Oral and Maxillofacial Surgery, University of Groningen and University Medical Center Groningen, the Netherlands; 4 Department of Medical Microbiology, University of Groningen, University Medical Center Groningen, the Netherlands

## Abstract

Periodontitis is an infective process that ultimately leads to destruction of the soft and hard tissues that support the teeth (the periodontium). Periodontitis has been proposed as a candidate risk factor for development of the autoimmune disease rheumatoid arthritis (RA). *Porphyromonas gingivalis*, a major periodontal pathogen, is the only known prokaryote expressing a peptidyl arginine deiminase (PAD) enzyme necessary for protein citrullination. Antibodies to citrullinated proteins (anti-citrullinated protein antibodies, ACPA) are highly specific for RA and precede disease onset. Objective of this study was to assess *P. gingivalis* PAD (PPAD) gene expression and citrullination patterns in representative samples of *P. gingivalis* clinical isolates derived from periodontitis patients with and without RA and in related microbes of the *Porphyromonas* genus. Our findings indicate that PPAD is omnipresent in *P. gingivalis,* but absent in related species. No significant differences were found in the composition and expression of the PPAD gene of *P. gingivalis* regardless of the presence of RA or periodontal disease phenotypes. From this study it can be concluded that if *P. gingivalis* plays a role in RA, it is unlikely to originate from a variation in PPAD gene expression.

Periodontitis is an infective process that ultimately leads to the destruction of the soft and hard tissues that support the teeth (the periodontium). Periodontitis has been proposed as a candidate risk factor for rheumatoid arthritis (RA)^1^. One of the biologically plausible causal mechanisms accounting for the association between periodontitis and RA could be induction of RA-related autoimmunity at inflamed mucosal sites, e.g. the periodontium^2^.

Antibodies against citrullinated proteins (ACPA) are highly specific (98%) for RA^3^ and can precede the clinical onset of RA^4^. Citrullination is a post-translational modification catalyzed by a family of enzymes called peptidylarginine deiminases (PAD)^5^. In this reaction, an arginine residue within a protein is converted into the non-coded amino acid citrulline. This modification leads to a loss of positive charge, reduction in hydrogen-bonding ability and subsequently in conformational and functional changes of the protein.

*Porphyromonas gingivalis* is a major periodontal pathogen involved in destructive periodontal disease^6^ and is the only known prokaryote expressing a PAD enzyme^7^. *P. gingivalis* PAD (PPAD) is both a secreted and a cell or membrane vesicle associated enzyme^7^. In contrast to human PAD, PPAD is able to modify free arginine and is not dependent on calcium^7^,^8^. Citrullination by PPAD enhances the survivability and increases the fitness of *P. gingivalis* due to several immune defense mechanisms. Additionally, a side effect of citrullination is ammonia production, which has a negative effect on neutrophil function and is protective during the acidic cleansing cycles of the mouth^7^,^8^. PPAD is regarded as a virulence factor because citrullination by PPAD interferes with complement activity^9^, inactivates epidermal growth factors^10^ and contributes to infection of gingival fibroblasts and induction of the prostaglandin E2 synthesis^11^. Moreover, PPAD has been reported to be able to generate citrullinated forms of various arginine-containing proteins and peptides^8^, among which are human fibrinogen and human α-enolase, two candidate auto-antigens in RA^12^.

A role of PPAD in autoimmunity is conceivable, considering that citrullinated host peptides generated by *P. gingivalis* are likely to expose epitopes previously hidden to the immune system, which may trigger an immune response in a genetically susceptible host^13^. In fact, cross reactivity has been shown for human antibodies against recombinant CEP-1, an immunodominant epitope of human α-enolase, with *P. gingivalis* enolase^14^. Moreover, there is strong animal experimental evidence supporting the theory that PPAD is the key player linking periodontitis and arthritis^15^,^16^.

Whether expression of PPAD is ubiquitous in *P. gingivalis* and whether there are different forms of the gene among *P. gingivalis* isolates from clinically different donors is currently unknown. Among oral bacteria, citrullination of endogenous proteins has only been shown in the *P. gingivalis* wild-type strain W83 and four clinical isolates from patients with periodontitis without RA^12^. Related species such as *Porphyromonas endodontalis*, indigenous to the oral cavity, and *Porphyromonas asaccharolytica,* commonly found in the gastrointestinal tract, have not been tested for citrullination capacity.

The aim of this study was to assess expression of the PPAD-encoding gene in representative samples of *P. gingivalis* clinical isolates from patients with and without RA, as well as in related species of the genus *Porphyromonas* and in the periodontal pathogens *Prevotella intermedia* and *Fusobacterium nucleatum*. Additionally, variation in gene composition was analyzed using a combination of primer sets for the whole gene and for a region including the active site of the gene, by restriction enzyme analysis of the PCR products with three different restriction enzymes, and by whole gene sequencing. Functional analysis of PPAD was carried out by assessment of endogenous citrullination patterns.

## Methods

### PPAD

#### Bacterial strains and culture conditions

Twelve *P. gingivalis* strains were isolated from 12 consecutive patients with RA and periodontitis, participants of an observational study on periodontitis and RA^17^. Eighty *P. gingivalis* strains were isolated from 80 consecutive subjects without RA (non-RA) with various periodontal diagnoses (periodontitis (n = 75), peri-implantitis (n = 2), gingivitis (n = 1) or a healthy periodontium (healthy carriers, n = 2), recruited for the control group of the same observational study^17^. This study was approved by the Medical Ethics Committee of the University Medical Center Groningen (METc UMCG 2011/010), and conducted in accordance with the guidelines of the Declaration of Helsinki and the institutional regulations. Written informed consent was obtained from all patients. Of note, this study only involved the collection of bacteria; the actual experiments did not involve human subjects and no tissue samples were used. Some general characteristics of the subjects from whom *P. gingivalis* was isolated are listed in Table 1. These clinical isolates, the *P. gingivalis* reference strains ATCC 33277 and W83, *P. asaccharolytica* (clinical isolate), *P. endodontalis* (clinical isolate), *F. nucleatum* (ATCC 25586) and *P. intermedia* (clinical isolate) were anaerobically grown on blood agar plates (Oxoid no. 2, Basingstoke, UK) supplemented with sheep blood (5% v/v), hemin (5 mg/l) and menadione (1 mg/l) and incubated in 80% N_2_, 10% H_2_ and 10% CO_2_, at 37 °C^6^.

#### DNA extraction

Colonies from a blood agar plate were suspended in 500 μL Tris-EDTA buffer and bacterial DNA was isolated utilizing a Precellys®24 Technology tissue homogenizer (Bertin Technologies) (3 times per 30 sec. at 5000 rpm with 30-sec. breaks in between). Afterwards, samples were boiled for 10 min. at 95 °C and centrifuged at 16100 g, 4 °C for 10 min. Supernatants were collected and stored at −20 °C. For whole genome sequencing, total DNA was extracted from 7 *P. gingivalis* strains using the Ultraclean Microbial DNA Isolation Kit (MO BIO Laboratories, Carlsbad, CA, US) following the manufacturer’s instructions.

#### PPAD PCR

PCR was performed on the PPAD gene using Phusion DNA Polymerase (Thermoscientific) and two sets of primers. The first pair, P1F and P1R, covered the whole gene and the second pair, P2F and P2R, covered a short region around the active site (Cys351). The sequence of P1F was 5′-GGGGAGCTCATGAAAAAGCTTTTACAGGCTAAAGC-3′ while the sequence of P1R was 5′-GGGCTCGAGTTTGAGAATTTTCATTGTCTCACGG-3′. The sequence of P2F was instead 5′-CTGATTCTGAACAACAGGGT-3′, while the sequence of P2R was 5′-TAAAGCTACCGGAACCATTG-3′. The samples were denatured at 98 °C for 10 seconds, annealed at 56 °C for 20 seconds and extended at 72 °C for 2 minutes for a total of 33 cycles. Analysis was then performed using gel electrophoresis on a 1% agarose gel, immersed in SB buffer (10 mM NaOH; 36 mM boric acid, pH 8.0) and subjected to 120 V for 30 minutes.

#### Restriction enzyme analysis

DNA samples for restriction enzyme analysis were cleaved with the four-nucleotide cutters *Sau*3AI, *Taq*αI or *Dpn*I following the instructions of the supplier (New England Biolabs) (incubation for 90 minutes at 37 °C for *Sau*3AI and *Dpn*I and at 65 °C for *Taq*αI, followed by heat inactivation for 20 minutes at 80 °C for *Taq*αI and *Dpn*I and at 65 °C for *Sau*3AI). *Sau*3AI and *Taq*α recognize the same sequence (GATC) but cut at different positions while *Dpn*I recognizes TCGA.

#### Whole genome sequencing and data analysis

DNA was extracted from a representative random sample of 7 *P. gingivalis* isolates that had been obtained from two RA patients with severe periodontitis, two RA patients with moderate periodontitis, two non-RA patients with severe periodontitis and one healthy carrier. The isolates originated from unrelated individuals. The DNA concentration and purity were controlled by a Qubit® 2.0 Fluorometer using the dsDNA HS and/or BR assay kit (Life technologies, Carlsbad, CA, US). The DNA library was prepared using the Nextera XT -v3 kit (Illumina, San Diego, CA, US) according to the manufacturer’s instructions and then run on a Miseq (Illumina) for generating paired-end 300 bp reads. *De novo* assembly was performed with CLC Genomes Workbench v7.0.4 (Qiagen, Hilden, Germany) after quality trimming (Qs ≥ 20) with optimal word size^18^. PPAD gene sequences were derived from the 7 assembled genomes and from 5 *P. gingivalis* genomes retrieved from GenBank (accession: NC_002950, NC_010729, NC_015571, CP007756 and AJZS01). DNA and amino acid sequences of 12 PPAD genes were aligned using the MAFFA v7 web server (http://mafft.cbrc.jp/alignment/software/). The PPAD gene sequences of strains 20655, 20658, MDS-16, MDS-45, MDS-56, MDS-85 and MDS-140 have been deposited at DDBJ/EMBL/GenBank under the accession numbers KP862650-KP862656.

### Endogenous protein citrullination patters

#### Bacterial strains and culture conditions

The 12 *P. gingivalis* isolates from patients with RA and 12 randomly selected *P. gingivalis* isolates from non-RA subjects, and individual clinical isolates of *P. asaccharolytica, P. endodontalis* and *F. nucleatum* were analyzed for endogenous protein citrullination patterns. The isolated bacterial strains were anaerobically grown on blood agar plates (Oxoid no. 2, Basingstoke, UK), which were supplemented with sheep blood (5% v/v), hemin (5 mg/L) and menadione (1 mg/L) and incubated in 80% N_2_, 10% H_2_ and 10% CO_2_, at 37 °C.

#### Bacterial cell lysate preparation

Four-day old colonies of monocultures of the selected bacterial strains were suspended in sterile phosphate buffered saline (PBS) with protease inhibitors (Complete Mini Protease Inhibitor Cocktail Tablets, Roche Diagnostics, 1 tablet for 7 ml PBS). After washing and centrifugation cycles (3 × 5 min., 14489 g, 4 °C) the bacterial pellets were resuspended in lysis buffer containing non-denaturing detergent (Noninet P-40, Sigma-Aldrich, Inc.) and sonicated on ice for 15 min. (Bioruptor® Standard sonication device, Diagenode s.a.). Protein concentration was determined using the BCA Protein Assay Kit (Thermo Scientific, Pierce Protein Biology Products).

#### SDS-PAGE and gel staining

Bacterial cell lysates were prepared with 2× SDS sample buffer (4% SDS, 20% glycerol, 10% β-mercaptoethanol, 125 mM Tris-HCl (pH 6.8) and 0.02% bromophenol blue) and boiled for 5 min. Per sample, 15 μg of protein was loaded onto a 12.5% SDS-PAGE gel (Criterion Tris-HCl, Bio-Rad Laboratories, Inc.) and resolved by running at 200 V and 15 Watt constant for 1.5 hours. Gels were stained using Coomassie® staining (SimplyBlue™ SafeStain, Life Technologies Corporation) or transferred to a PVDF membrane (Immobilon® EMD Millipore Corporation, Billerica, MA, USA).

#### Western Blot

Citrulline-containing proteins were detected by Western blotting with a polyclonal IgG antibody (Anti-Citrulline Modified Detection Kit, Upstate, EMD Millipore Corporation, Billerica, MA, USA) according to the manufacturer’s instructions. In addition, detection of citrulline containing proteins was done with a monoclonal IgM antibody (F95) against a deca-citrullinated peptide (U2005-0033, UAB Research Foundation, Birmingham AL) using the following protocol: after blocking for one hour using a 1:1 dilution of Odyssey® Blocking Buffer (LI-COR Biosciences, Lincoln, USA) and PBS, incubation with F95 in the same blocking buffer (final dilution 1:2000) with 0.1% Tween^®^20 (Sigma-Aldrich Co. LLC.) was done overnight at 4 °C. IRDye® 800 conjugated goat anti-mouse IgM (Rockland Immunochemicals Inc., Gilbertsville) (1:10000) in Odyssey® Blocking Buffer and PBS (1:4) with 0.1% Tween^®^20 (Sigma-Aldrich Co. LLC.) was used as secondary antibody for one hour at room temperature. *In vitro* citrullinated human fibrinogen (341578, Calbiochem, distributed by VWR international) by rabbit PAD (P1584, Sigma-Aldrich Co. LLC.) was used as positive control^19^. Non-specific binding of the secondary antibody was excluded by omitting the primary antibody. Protein bands were detected by the Odyssey system (LI-COR Biosciences, Lincoln, USA). For graphical reproduction of the gels, the signal and size of the protein bands were analyzed using Image Studio Version 2.0.38 (LI-COR Biosciences, Lincoln, USA) with the same image display settings per gel. *F. nucleatum* was considered as negative control^12^ and, if present, the signal of the detected bands was corrected for the mean signal of *F. nucleatum*. The sizes of detected bands were plotted in a graph using GraphPad Prism 5 (GraphPad Software Inc.).

## Results

### PPAD gene is a conserved feature of *P. gingivalis*

**Figure 1:**
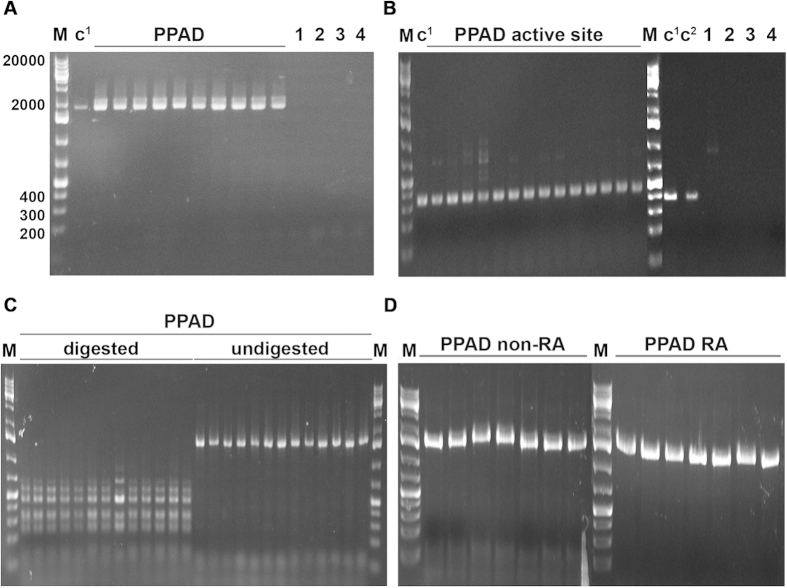
PPAD gene composition analyzed by PCR and restriction enzyme analysis of the PCR products. (**A**) PCR products of PPAD obtained with whole-gene primers (1668 base pairs) using 10 representative *P. gingivalis* isolates of patients without RA. No PPAD genes are detectable in other *Porphyromonas* species or other selected periodontal pathogens. (**B**) PCR products of PPAD obtained with active site region primers (328 base pairs) of 14 representative *P. gingivalis* isolates from patients without RA. No PPAD genes are detectable in other *Porphyromonas* species or other selected periodontal pathogens. (**C**) Restriction enzyme analysis with *Sau*3AI of PPAD PCR products obtained with whole-gene primers of 13 representative *P. gingivalis* isolates from patients without RA. (**D**) PPAD PCR products obtained with whole-gene primers of 14 representative *P. gingivalis* isolates from patients with or without RA. **M** = marker displayed as number of base pairs (GeneRuler™ 1 kb Plus DNA ladder). **C**^**1**^ = positive control (PPAD of *P. gingivalis* W83). **C**^**2**^ = positive control (PPAD of *P. gingivalis* ATCC 33277). **1** = *P. intermedia*, **2** = *P. asaccharolytica*, **3** = *P. endodontalis* and **4** = *F. nucleatum.* **Digested** = PPAD PCR products digested with *Sau*3AI. **Undigested** = PPAD PCR products of the same 13 *P. gingivalis* clinical isolates not incubated with *Sau*3AI. **non-RA** = without rheumatoid arthritis. **RA** = with rheumatoid arthritis.

The PPAD gene, consisting of 1668 base pairs, was detected by PCR in all 92 investigated *P. gingivalis* strains, but not in any of the other bacterial species tested (Fig. 1A). The same holds true for the region encoding the active site of PPAD, consisting of 328 base pairs (Fig. 1B). Cleavage of the PCR-amplified PPAD genes with three different restriction endonucleases and subsequent separation of the fragments by gel electrophoresis revealed no differences in the respective banding patterns for all 92 investigated *P. gingivalis* strains (shown for *Sau*3AI in Fig. 1C). Furthermore, no differences in the whole PPAD gene or in the active site-encoding regions of PPAD were observed between *P. gingivalis* isolates from RA patients or *P. gingivalis* isolates from non-RA patients (shown for the whole PPAD gene in Fig. 1D).

### Conservation of PPAD gene sequence

Alignment of the PPAD gene sequences of the *P. gingivalis* strains revealed that the PPAD gene is highly conserved among all analyzed strains. At the DNA and amino acid level, no mutations were found in the signal peptide region and also the active site Cys351 residue is strictly conserved. Overall, the PPAD protein of each strain analyzed has no more than five different amino acids compared to the PPAD proteins of the reference strains W83 or ATCC 33277. In addition, none of the mutations is an insertion, deletion or leads to proteins truncations. However, allelic differences were detected in the PPAD gene sequences, especially for the clinical isolates 20655 (derived from a non-RA patient with periodontitis) and MDS-85 (derived from an RA patient with periodontitis), which displayed 23 and 18 single nucleotide mutations respectively compared to the reference strain W83. Table 2 summarizes the number of nucleotides in the PPAD genes and amino acid residues in the PPAD proteins that differentiate each strain from any other.

Interestingly, the highest number of identified mutations is 25, which separates the PPAD genes from isolates 20655 and 20658 (both derived from non-RA patients with severe periodontitis), and from isolates 20655 (derived from a non-RA patient with severe periodontitis) and MDS-140 (derived from a healthy carrier). Notably, besides their low numbers, the majority of these mutations were synonymous. Taken together, these results show a very high level of PPAD conservation in the investigated *P. gingivalis* isolates.

### Endogenous protein citrullination patterns

**Figure 2:**
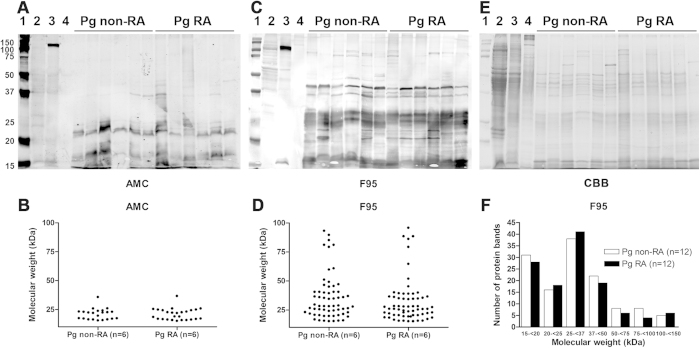
Patterns of citrullinated proteins of *P. gingivalis* isolates from patients with or without RA. (**A,C,E**) Western blots and Coomassie staining of bacterial cell lysates of 12 representative *P. gingivalis* isolates of patients with or without RA (both n = 6). (**A**) Citrullinated protein patterns as detected with the AMC detection method (AMC). (**C**) Citrullinated protein patterns as detected with the F95 anti-citrulline antibody (F95). (**E**) Coomassie staining. (**B,D**) Graphical representation of the Western blots shown in panels (**A,C**). (**B**) Citrullinated protein patterns as detected with the AMC detection method (AMC). (**D**) Citrullinated protein patterns as detected with the F95 anti-citrulline antibody (F95). (**F**) Graphical representation of citrullinated protein patterns as detected by Western blots using the F95 anti-citrulline antibody (F95) against bacterial cell lysates of 24 representative *P. gingivalis* isolates of patients with or without RA (both n = 12). The Western blots were analyzed with the same image display settings. **1** = Molecular weight marker in kilo Dalton (kDa), **2** = *P. asaccharolytica,* **3** = *P. endodontalis,* **4** = *F. nucleatum,* **Pg non-RA** = *P. gingivalis* isolates from subjects without RA, **Pg RA** = *P. gingivalis* isolates of patients with RA. The strong positive staining at circa 120 kDa in *P. endodontalis* (3) both with the AMC and the F95 detection method (panels A,C) is due to non-specific binding of the secondary antibody.

To determine possible differences in the protein citrullination activities of different PPAD enzymes both the AMC detection method and the anti-citrulline F95 monoclonal antibody were employed. Both assays showed that the patterns of citrullinated proteins of *P. gingivalis* isolates from patients with RA were not detectably different when compared to the pattern of citrullinated proteins from *P. gingivalis* isolates from non-RA patients. Figure 2 (panels A,C,E) shows the Coomassie-stained gel and Western blots for 6 representative *P. gingivalis* isolates of each group, including a graphical representation of the respective citrullination patterns (panels B,D). After correction for conjugate controls*, P. asaccharolytica* and *P. endodontalis* showed no protein bands with the AMC detection method. However, some citrullinated protein bands were observed for these species when the F95 antibody was applied. Neither of the two detection methods revealed citrullinated proteins in samples of *F. nucleatum* (Fig. 2, panels A,C).

## Discussion

This is the first study assessing PPAD expression in a large sample of clinical *P. gingivalis* isolates obtained from patients with or without RA. Our findings indicate that PPAD is omnipresent in *P. gingivalis,* but absent from *P. endodontalis* and *P. asaccharolytica* as well as from the other periodontal pathogens studied.

Our present observations support the view that PPAD may represent one of few, if not the only prokaryotic peptidylarginine deiminase. Of note, our analyses show that the PPAD gene is highly conserved in *P. gingivalis.* Consequently, the encoded PPAD enzymes share 98.9–100% amino acid sequence identity. This may suggest that PPAD contributes to the ability of *P. gingivalis* to colonize and thrive in its human host. Notably, some mutations in PPAD are missense and it may be of interest to analyze the citrullination levels of the respective PPAD isotypes, in order to see whether these mutations influence the enzymatic activity. Similarly, the mammalian PAD enzyme is also highly conserved with 70–95% identical amino acids sequences^5^; hinting at the importance of protein citrullination for both mammals and *P. gingivalis*, although we found no indications that the PPAD is evolutionarily related to the mammalian PAD enzymes^20^.

Concerning PPAD, no differences were noted in the PPAD genes among *P. gingivalis* isolates from patients with or without RA. Also, no differences in PPAD genes were noted among *P. gingivalis* isolates from patients with different stages of periodontal disease or periodontal health. Therefore, we assume that there are no different PPAD variants in *P. gingivalis.* Functional analysis of PPAD further substantiated this assumption. No differences in endogenous citrullination patterns were seen between *P. gingivalis* isolates from RA and non-RA patients, as determined with two different anti-citrulline antibodies. Some differences were observed in the citrullination patterns detected with the two antibodies against citrullinated proteins, which can probably be attributed to the monoclonal (F95) or polyclonal (AMC) nature and the isotypes of these antibodies (IgM and IgG, respectively). Another difference between the antibodies is the chemical modification of the citrulline residues in the AMC detection method to ensure detection of citrulline-containing proteins regardless of neighboring amino acid sequences.

Based on the observations in this study, we conclude that PPAD is apparently omnipresent in *P. gingivalis* but absent from *P. asacharolytica* and *P. endodontalis*, two related species of the genus *Porphyromonas.* There are no significant differences in the PPAD gene regardless of RA or periodontal disease phenotypes. Therefore, from this study it can be concluded that if *P. gingivalis* plays a role in RA, it is unlikely to originate from a variation in PPAD gene expression.

An important future goal to strive for will be a detailed characterization of the function of the PPAD protein and its post-translational modifications. The production of recombinant PPAD in *Escherichia coli* has been studied in order to investigate its protein function. The catalytic mechanism was identified and showed different enzyme activities based on an N-terminal truncation of the protein^21^. This finding is in accordance with a recent study by Konig *et al.* which showed that non-cleaved PPAD is autocitrullinated and has decreased activity^22^. Additionally, Konig *et al.* concluded that autocitrullination of PPAD is not the underlying mechanism linking *P. gingivalis* with RA because it does not occur in *P. gingivalis* cells and patient antibodies were directed specifically against non-citrullinated PPAD. Conversely, another recent study showed a peptidyl-citrulline specific antibody response in patients and concluded that PPAD autocitrullination is still a potential mechanism for breaching autoimmunity in RA patients^20^. Besides these theories mainly focusing on cleavage and autocitrullination of PPAD, it will be crucial to investigate the overall citrullination of bacterial and host proteins by PPAD in especially the *in vivo* situation, as well as the interaction of the human PADs with bacterial proteins, as proposed by Quirke *et al.*^20^. In conclusion, it is more likely that a difference in post-translational modification of PPAD might play an important role in RA, rather than a difference in the PPAD gene.

## Additional Information

**How to cite this article**: Gabarrini, G. *et al.* The peptidylarginine deiminase gene is a conserved feature of *Porphyromonas gingivalis. Sci. Rep.* **5**, 13936; doi: 10.1038/srep13936 (2015).

## Figures and Tables

**Table 1:** General characteristics of subjects from whom *P. gingivalis* was isolated.

Patient group	number	median age(years, IQR)	currentsmoker (%)	female(%)
RA	12	64 (56–71)	25	75
non-RA	80	51 (42–60)	27	54
**Periodontal diagnosis**	**RA (n)**	**non-RA (n)**		
periodontitis	10	75		
peri-implantitis		2		
gingivitis	2	1		
healthy		2		
*Characteristics of RA patients				
median disease duration (months, IQR)	37 (27–109)			
median DAS28 (IQR)	2.3 (1.6–4.0)			
median CRP (mg/l, IQR)	3 (3–14)			
anti-CCP seropositive (%)	92			
IgM-RF seropositive (%)	92			
MTX monotherapy (%)	92			

**RA:** rheumatoid arthritis, **non-RA:** without rheumatoid arthritis, **IQR**: interquartile range, **n:** number, **DAS28:** disease activity score 28 tender and swollen joint count, **CRP:** C-reactive protein, **anti-CCP**: anti-cyclic citrullinated protein antibody, **IgM-RF:** IgM rheumatoid factor, **MTX:** methotrexate,

^*^for details see^17^.

**Table 2:**
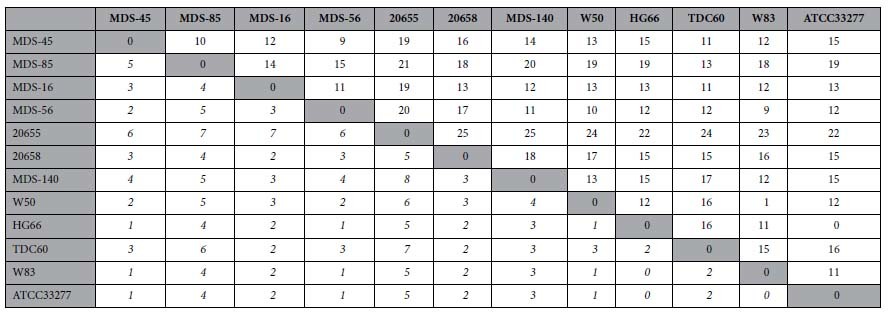
Representation of the numbers of different nucleotides in PPAD genes and numbers of amino acid substitutions in the corresponding PPAD proteins.

Top right, number of different nucleotides; bottom left, number of amino acid substitutions (italic). PPAD gene sequences from *P. gingivalis* isolates obtained from two RA patients with severe periodontitis (**MDS-45, MDS-85**), two RA patients with moderate periodontitis (**MDS-16, MDS-56**), two non-RA patients with severe periodontitis (**20655, 20658**) and one healthy carrier (**MDS-140**). Additional PPAD gene sequences were retrieved from GenBank (**W50, HG66, TDC60, W83, ATCC 33277**).
